# A Novel Trace Element Mixture to Enhance Growth, Blood and Carcass Characteristics of Growing Rabbits

**DOI:** 10.1007/s12011-025-04897-3

**Published:** 2025-11-25

**Authors:** Mohamed I. Hassan, Amr El-Nile, Saber S. Hassan, Mohamed Hafez, Abdallah E. Mohamed, Alexander I. Popov, Mohamed Rashad, Sobhy M. A. Sallam, Mahmoud Alagawany

**Affiliations:** 1 Livestock Research Department, Arid Lands Cultivation Research Institute, City of Scientific Research and Technological Applications (SRTA-City), New Borg El-Arab, Alexandria, 21934 Egypt; 2 Animal and Poultry Production Department, Faculty of Agriculture, Damanhour University, Damanhour, Egypt; 3 Soil and Water Technologies Department, Arid Lands Cultivation Research Institute, City of Scientific Research and Technological Applications (SRTA-City), New Borg El-Arab, Alexandria, 21934 Egypt; 4 Department of Soil Science and Soil Ecology, Institute of Earth Sciences, Saint Petersburg State University, Embankment, 7/9, Saint Petersburg, 199034 Russia; 5 Department of Animal and Fish Production, Faculty of Agriculture, University of Alexandria, Alexandria, Egypt; 6 Department of Animal and Poultry Production, College of Agriculture and Food, Qassim University, Al-Qassim, Saudi Arabia Buraydah 51452,; 7 Department of Poultry, Faculty of Agriculture, Zagazig University, Zagazig, 44511 Egypt

**Keywords:** V-line, Growth performance, Hematology, Protein profile, Lipid profile

## Abstract

Animal producers face a series of problems; one of them is the availability of a high-quality product as a source of trace elements. The aim of this research study was to evaluate the effects of water supplementation of a Novel Trace Element Mixture (NTEM) on growth rate, blood, and carcass characteristics of growing rabbits. Seventy-two rabbits at seven weeks of age were used in the current study. Rabbits were randomly assigned to four treatment groups, each of six replicates (*n* = 18): control (0 NTEM), second, third, and fourth groups were supplemented with different levels of the NTEM (0.5, 1, and 1.5 mL/L, respectively) in the drinking water twice a week for five weeks. All groups were kept under the same managerial and environmental conditions. The results showed that adding NTEM at different levels to drinking water significantly enhanced body weight, body weight gain, feed conversion ratio, and the rabbit’s performance index compared with the control group. The obtained data showed that serum, hemoglobin, and red blood cell values were within optimal ranges at the end of the study in the NTEM treatment groups, except for the low level. In conclusion, supplementation of NTEM in drinking water at the tested levels improved growth performance, feed efficiency, and selected hematological and biochemical parameters in growing rabbits, without adverse effects on health or carcass traits.

## Introduction

Optimizing growth and health in growing rabbits is a critical goal in commercial and research settings, as it directly affects productivity, feed efficiency, and overall animal welfare. Trace elements such as zinc, copper, manganese, and selenium play essential roles in enzymatic reactions, antioxidant defense, immune function, and nutrient metabolism. Trace elements are essential micronutrients in livestock diets, as their availability directly affects health, growth efficiency, and overall production performance [36]. This makes their role of particular interest to producers, feed manufacturers, veterinarians, and animal scientists. [17].

The Fe^2+^ is the most abundant trace metal in mammals, present in haemoglobin, myoglobin, and a cofactor for many essential Fe-dependent enzymes, such as peroxidases and catalases. Around 70% of the total Fe body pool is composed of haemoglobin. Homeostasis tightly controls Fe absorption and retention in the intestine [21]. The Fe^2+^ content in biological systems exists in one of two oxidation states, as redox interconversions between ferrous (Fe^2+^) and ferric (Fe^3+^) are critical to the biological properties of this trace element. Fe^2+^ is required for aerobic metabolism. It participates in the transport of O_2_ and CO_2_ as a component of hemoglobin. It serves as an electron carrier and a cofactor for the majority of the Krebs cycle enzymes [20, 33], [26], [34].

Zn^2+^ is an essential cofactor for many enzymes involved in nucleic acid biosynthesis and cell division. It also enhances physiological functions like acid–base balance, metabolism, and immune defence [13]. Zn^2+^ is frequently used in many livestock diets to improve growth performance [38]. Zn’s antioxidant role and participation in antioxidant defense systems are among its most important functions [5].

Mn^2+^ is one of the essential trace elements for rabbits and is involved in many metabolic processes, including energy and protein metabolism, and skeletal, reproductive, and immune system development [34]. Mn^2+^ is an essential and basic micro-element that is naturally present in many foods and drinks, as well as in formula, parenteral nutrition, and supplements [25]. Additionally, it is a vital trace element found in all organic tissues and is essential for the normal metabolism of amino acids, lipids, proteins, and carbohydrates. Moreover, Mn^2+^ is essential for healthy immune function, as well as for controlling blood sugar, energy, reproduction, digestion, and bone formation. It also plays a role in numerous organ systems. [3].

Cu^2+^ is one of the many essential elements in an increasing variety of cuproenzymes and copper metalloproteins, which perform diverse functions, such as electron transfer [12]. Although Cu^2+^ is not a necessary part of hemoglobin, it is found in a few other plasma proteins, including ceruloplasmin, which regulates the release of Fe from cells into the plasma [19]. Zn and Cu are antagonists, and biological dualism is an example of the balance between them. This does that, and that does this, and they frequently fight. Both elements have critical roles in the body [39].

Boron (B) is a potentially useful and required element for higher animals and humans, according to research from multiple laboratories employing a range of experimental models. B is reported to have favourable effects on bone maintenance and growth [15]. B also enhances the biological processes of both humans and animals, including development, reproduction, energy metabolism, immunity, brain function, calcium metabolism, bone formation, and the steroid hormones oestrogen and vitamin D [23]. Additionally, B modulates parahormone activity, which affects the metabolism of calcium, magnesium, phosphorus, and cholecalciferol [18].

In conclusion, the trace elements (Fe^2+^, Zn^2+^, Mn^2+^, Cu^2+^, and B^+^) are essential micronutrients for animal and rabbit feeding, as they are involved in various metabolic processes and are required for maintaining optimal health and productivity. Also, the feed intake is the primary source of trace elements for animals, and it is important to ensure that the feed contains appropriate levels of these elements [30].

Previous studies have demonstrated that dietary supplementation with individual trace elements can improve growth performance, hematological parameters, and carcass traits in rabbits and other livestock. However, most studies focused on single elements or conventional mineral sources, and little is known about the synergistic effects of a well-formulated mixture of trace elements delivered through drinking water. Therefore, the objectives of this study were to evaluate the impact of different levels of NTEM on growth performance, hematological parameters, protein and lipid profiles, and carcass characteristics in V-Line rabbits. The findings aim to provide practical insights for improving rabbit production and to contribute to understanding trace element interactions in animal nutrition.

## Materials and Methods

### Trace elements mixture solution preparation

The following steps were taken to produce a Novel Trace Element Mixture (NTEM) solution: Fe-EDTA, aqueous solution, 1.84%, was mixed with 1L of distilled water, then ZnSO_4_•7H_2_O—0.29 g; MnSO_4_•H_2_O—1.7 g; CuSO_4_—0.8 g; H_3_BO_3_—1.8 g were added and mixed to the same solution. The solution was adjusted to pH 7.0 by adding phosphoric acid (10%) [14].

### Experimental Animals and Management

The study was conducted at El-Bostan Experimental Station, Faculty of Agriculture, Damanhour University, Beheira, Egypt. Rabbits were housed individually in wire cages (35 × 40 × 50 cm) under uniform environmental and management conditions. Feed was provided ad libitum, and clean water was available at all times. A total of seventy-two weaned male V-Line rabbits, seven weeks old and weighing 931.67 ± 3.19 g on average, were used in the experiment. All animals were obtained from the Poultry Research Centre, Faculty of Agriculture, Alexandria University, Egypt. The research study continued for five weeks. Four groups of rabbits were randomly assigned (*n* = 18), with six replicates per group, each with three rabbits per replicate. The animal groups were as follows: a control group receiving no NTEM supplementation, second, third, and fourth groups: varied concentrations of NTEM (0.5, 1, and 1.5 mL/L, respectively), administered twice weekly for five weeks. Throughout all treatments, all rabbits received identical basal diets (Table 1) formulated in accordance with the National Research Council (NRC, 1977).

### Performance traits

Feed consumption was calculated by subtracting the weight of feed leftovers from the total amount provided. Cumulative body weight, body weight gain, and feed conversion ratio were determined from collected data throughout the experimental period. At the end of the experiment, the performance index was calculated as follows:

$$\text{Performance index }\left({\%}\right)=\frac{\mathrm{BW}}{\mathrm{FCR}}\times 100$$

where: BW is cumulative body weight; FCR is feed conversion ratio [24].

### Blood parameters

Blood samples from the slaughtered rabbits were taken aseptically at the end of the research study (*n* = 6 rabbits for each group) and inserted into sterile tubes. The hematological traits were measured according to [28]. Red and white blood cells (RBC, WBC),hemoglobin and lymphocytes (Hb, LYM); packed cell volume (PCV); monocytes (MON); eosinophils (ESIN); basophils (BASO), and heterophils (HTERO) in the whole blood samples, were evaluated using an Auto Hematology Analyzer (Bioevopeak, US).

Each animal received a separate, sterile tube for the analysis of serum metabolites. The serum was then separated by centrifuging the tubes for 15 min at 3500 rpm after they had coagulated at room temperature for 30 min. The serum samples were stored at—20°C until use.

To determine the protein and lipid changes during the fattening period, the serum was analyzed. Serum total protein (g/dL), albumin (g/dL), total lipids, triglycerides, total cholesterol, high-density lipoprotein, and low-density lipoprotein were spectrophotometrically analyzed using commercial diagnostic kits from Diamond Diagnostics (23 EL-Montazah St., Heliopolis, Cairo, Egypt) on a spectrophotometer (Beckman DU-530, Germany). The difference between total protein and albumin was used to calculate the serum globulin level (g/dL), as fibrinogen typically accounts for only a small portion of total protein. [32]. The following globulin fractions: alpha, beta, and gamma globulins (α-, β-, and γ-, respectively) were identified. The entire amount of the overall serum protein concentration (g/dL) was used to determine the fractions’ quantities.

### Carcass characteristics

After the end of the research experiment (twelve weeks of age), after a 12-h fast, six rabbits from each treatment were randomly selected, weighed, and exsanguinated. Then, to prepare the carcass for study, the urinary bladder, genital organs, skin, feet, and paws were all cut off. The fresh carcass components, including the heart, kidneys, lungs, liver, and spleen, were weighed, categorized, and the relative weight from the hot BW was estimated and reported as g/kg of pre-slaughter weight. The carcass and dressing percentages were calculated as follows:

1 $$\text{Carcass }({\%}) =\text{weight of carcass}\times \frac{100}{\mathrm{LBW}}$$

2 $$\text{Dressing }({\%}) =(\text{Weight of carcass }+\text{Giblets weight}) \times \frac{100}{\mathrm{LBW}}$$

where: LBW is live body weight at slaughter.

### Statistical analyses

All result data were subjected to a change study using the SPSS software package [31]. Data were analyzed by one-way ANOVA. Also, significant differences among means were determined using Duncan’s multiple comparisons. Range test at 0.05 level of significance [10]. .

**Table 1:** Feed ingredients and calculated chemical composition of the basal diet

Ingredients	(kg/ton)	Chemical composition (g/kg)	
Yellow corn	100.0	Dry matter	903.2
Barley	130.0	Organic matter	804.8
Molasses	30.0	Crude protein	172.4
Clover hay	395.0	Crude fibre	134.6
Wheat bran	150.0	Ether extract	28.00
Soybean meal	175.0	Nitrogen-free extract	569.8
Dicalcium phosphate	8.0	Ash	95.20
Limestone	5.0	Digestible energy (kcal/kg)	2464
Sodium chloride	3.0		
Vitamin and minerals mixture*	3.0		
DL-methionine	1.0		

^*^Provision per kg of diet: Vit. A,1200 IU; Vit.D3, 2500 IU; Vit. E, 10 mg; Vit. K3, 3 mg; Vit.B1, 1 mg; Vit.B2, 4 mg; Pantothenic acid, 10 mg; Nicotinic acid, 20 mg; Folic acid, 1 mg; Biotin, 0.05mg; Niacin, 40 mg; Vit.B6, 3 mg; Vit. B12, 20 mcg; Choline Chloride, 400 mg; Mn, 62 mg; Fe,44 mg; Zn, 56 mg; I, 1 mg; Cu, 5 mg, and Se, 0.01mg

## Results

### Performance of weaned rabbits

The effects of different NTEM levels on the parameters of growing rabbits are shown in Table 2. The experimental rabbit’s body weight (BW) range at five weeks of age was 925.33–938.67 g. Addition of NTEM levels significantly (*p* < 0.05) increased the BW of growing rabbits at twelve weeks of age compared to the control group. However, there were no significant differences in NTEM levels among the rabbit groups. On the other hand, body weight gain (BWG) at five to ten weeks of age in growing rabbits followed a similar trend to the final BW at twelve weeks of age in the NTEM groups. However, no significant (*p* < 0.05) differences were recorded among the different NTEM levels groups.

**Table 2:** Influence of NTEM on the growth performance of the rabbits during the experiment

Item	Control	NTEM^1^ (mL/L)			SEM^2^	P-value
		0.5	1.0	1.5		
Initial BW^3^ (g)	931.00	938.67	931.67	925.33	3.19	0.561
Final BW (g)	1895.00^b^	1976.30^a^	2009.00^a^	1998.00^a^	11.57	< 0.001
BWG^4^ (g)	964.00^b^	1037.70^a^	1077.00^a^	1072.70^a^	11.18	< 0.001
Feed consumption (g)	2965.30	2819.30	2872.30	2881.30	21.37	0.103
FCR^6^	3.08^a^	2.72^b^	2.67^b^	2.69^b^	0.04	< 0.001
performance index (%)	61.63^b^	72.83^a^	75.49^a^	74.40^a^	2.00	0.018

^a^^,^^b^ The different letters mean statistically significant at (*p* < 0.05); ^1^NTEM: Novel Trace Element Mixture, ^2^SEM: the standard error of mean, 3BW: body weight, 4BWG: body weight gain, 5FCR: feed conversion ratio

NTEM supplemented at the experimental levels significantly enhanced the feed conversion ratio (FCR) (2.67–2.72) and performance index (PI) (72.83–75.49%) against the control group (3.08 g/g and 61.63%) for a 7–12 weeks’ rabbit growing period, respectively (Table 2).

### Hematological parameters

The effects of NTEM on the hematological characteristics of growing rabbits are shown in Table 3. There are no significant differences in packed cell volume (PCV), white blood cells (WBC), lymphocytes (LYM), eosinophils (ESIN), and neutrophils (NEUT) values due to adding NTEM to V-Line rabbits compared to the control group. On the other hand, the 0.5 ml of NTEM significantly increased red blood cells (RBC) and hemoglobin (Hb) values compared with the control and the 1.5 ml of NTEM groups. In addition, each level of NTEM significantly (*p* < 0.05) increased the monocyte percentage (13.27–13.98%) compared to the control group (11.75%). However, no significant differences were observed between the different NTEM level groups for monocyte percentage. Furthermore, 0.5 ml of NTEM significantly decreased the rate of basophils (BASO) compared with the control and the 1.0 and 1.5 ml NTEM groups.

**Table 3:** Influence of NTEM on hematology of growing rabbits

Item^3^	Control	NTEM^1^ (mL/L)			SEM^2^	P-value
		0.5	1.0	1.5		
RBC (10^6^/µL)	1.63^b^	2.68^a^	2.22^ab^	1.60^b^	0.14	0.011
Hgb (g/dL)	9.03^b^	11.06^a^	9.66^ab^	8.68^b^	0.31	0.024
PCV (%)	39.85	37.50	39.39	42.88	0.81	0.118
WBC (10^3^/µL)	8.65	9.72	8.90	8.67	0.20	0.176
LYM (%)	44.46	42.97	43.03	43.41	0.35	0.425
MON (%)	11.75^b^	13.63^a^	13.27^a^	13.98^a^	0.25	0.002
ESIN (%)	11.64	11.95	12.51	11.40	0.21	0.259
BASO (%)	0.01^b^	0.67^a^	0.01^b^	0.01^b^	0.08	< 0.001
NEUT (%)	32.15	30.78	30.95	31.22	0.30	0.389

^a^^, b^ The different letters indicate statistical significance (*p* < 0.05). ^1^NTEM: novel trace element mixture, ^2^SEM: the standard error of mean, ^3^Red and white blood cells (RBC, WBC); hemoglobin and lymphocytes (Hb, LYM); packed cell volume (PCV); monocytes (MON); eosinophils (ESIN); basophils (BASO), and heterophils (HTERO)

### Protein profile

The results of adding NTEM to weaned V-Line rabbits on protein are given in Table 4. NTEM at 1.5 mL significantly increased serum albumin levels compared with all treatments. Meanwhile, both 0.5 and 1.5 mL of NTEM significantly decreased serum albumin concentration compared to the control group. NTEM and the control group had no significant effect on α-globulin (g/L), β-globulin (g/L), and γ-globulin (g/L).

**Table 4:** Influence of NTEM on the protein profile of rabbits

Item	Control	NTEM^1^ (mL/L)			SEM^2^	P-value
		0.5	1.0	1.5		
Total protein (g/L)	55.39^a^	54.68^a^	54.72^a^	51.25^b^	0.50	0.006
Albumin (g/L)	22.92^c^	26.05^b^	22.42^c^	28.28^a^	0.56	< 0.001
Globulin (g/L)	32.47^a^	28.64^b^	32.30^a^	22.97^c^	0.85	< 0.001
α- Globulin (g/L)	17.07	16.42	17.42	16.75	0.19	0.292
β- Globulin (g/L)	11.12	10.12	11.12	10.78	0.25	0.457
ɣ- Globulin (g/L)	7.50	7.41	8.01	6.00	0.29	0.083

^a^^,^^b, c^ The different letters mean statistically significant at (*p* < 0.05). ^1^NTEM: novel trace element mixture, ^2^SEM: the standard error of mean

### Lipid profile

The effect of NTEM on the lipid profile of growing rabbits is detailed in Table 5. V-Line rabbits receiving NTEM supplementation exhibited no significant changes in total lipids and cholesterol concentrations compared with the control group or among different levels of NTEM under investigation.

**Table 5:** Effect of NTEM on the lipid profile of rabbits

Item^3^	Control	NTEM^1^ (mL/L)			SEM^2^	P-value
		0.5	1.0	1.5		
Total lipid (g/dL)	60.32	53.65	60.99	57.76	1.31	0.184
Triglyceride (mg/dL)	103.12^a^	90.97^b^	76.01^c^	84.23^bc^	2.69	< 0.001
Cho (mg/dL)	39.90	38.25	39.52	35.09	1.04	0.366
HDL (mg/dL)	7.92^c^	9.56^ab^	8.86^b^	10.21^a^	0.22	< 0.001
LDL (mg/dL)	25.06^a^	18.60^c^	21.87^b^	25.95^a^	0.77	< 0.001

^a^^,^^b,c^ The different letters mean statistically significant at (*p* < 0.05). ^1^NTEM: novel trace element mixture, ^2^SEM: the standard error of mean, 3Cho: cholesterol, HDL: high density lipoprotein, LDH: low density lipoprotein

Moreover, the medium level of NTEM significantly (*p* < 0.05) reduced triglyceride concentration compared with a low level of NTEM and the control groups (Table 5). The highest high-density lipoprotein (HDL) concentration was observed in the NTEM group compared to the control group. In addition, the low and medium levels of NTEM significantly (*p* < 0.05) decreased low-density lipoprotein (LDL) compared with the other treatments.

### Carcass characteristics

The effect of NTEM on carcass characteristics as a percentage of the pre-slaughter weight of growing V-line rabbits is shown in Table 6. No significant effects were observed in most carcass traits due to adding NTEM to V-Line rabbits compared to the control group, except for liver weight percentage. It is noted that adding NTEM at a low level to rabbits significantly (*p* < 0.05) increased liver weight percentage compared to the other treatments.

**Table 6:** Influence of NTEM on carcass characteristics (as % of Pre-Slaughter Weight) of rabbits

Item (%)	Control	NTEM^1^ (mL/L)			SEM^2^	P-value
		0.5	1.0	1.5		
Carcass	56.57	53.20	56.46	54.94	0.55	0.067
Dressing	61.78	59.09	61.73	60.31	0.52	0.214
Giblets	5.21	5.89	5.28	5.36	0.13	0.276
Heart	0.34	0.34	0.41	0.37	0.01	0.118
Liver	3.39^b^	4.35^a^	3.68^b^	3.73^b^	0.13	0.023
Kidney	0.81	0.72	0.72	0.76	0.02	0.405
Lungs	0.67	0.49	0.47	0.50	0.05	0.565
Spleen	0.07	0.06	0.06	0.07	0.05	0.770

^a^^,^^b^ The different letters mean statistically significant at (*p* < 0.05). ^1^NTEM: novel trace element mixture, ^2^SEM: the standard error of mean

## Discussion

Supplementation of NTEM in drinking water improved growth performance in weaned rabbits, as evidenced by higher final body weight, weight gain, feed conversion ratio, and performance index compared with the control. This suggests that NTEM enhanced nutrient utilization and growth efficiency during the post-weaning period. The absence of significant differences among NTEM levels indicates that even the lowest dose was sufficient to achieve these benefits. In the current study, all NTEM treatment groups showed significant increases in final BW, BWG, and FCR. This effect may be explained by the NTEM components, which are more effective than the control diet at stimulating the activity of some growth factors. Our research supports the findings of [29], who stated that the activity of more than 200 metalloenzymes and biological processes in all living matter requires the inclusion of Zn. As a result, Zn is essential for development, appetite, skin health, and brain functions.

Additionally, because copper inhibits harmful intestinal microbes, it can accelerate growth and increase feed efficiency when added at levels above the normal requirement [2]. Moreover, B is regarded as a prebiotic chemical element, playing a role in the genesis and evolution of life and having positive effects on biological processes in both humans and animals, including reproduction, growth, calcium metabolism, bone formation, and energy metabolism [23]. Also, B plays a crucial role in stimulating the growth of beneficial bacteria and the production of short-chain fatty acids (SCFAs), which can interact with host tissues in various ways [7].

In the same context, [4] observed that average daily gain and the ratio of feed to BWG (F/G) in growing Rex rabbits were significantly impacted by the manganese supplementation group. The results indicated that blood albumin concentration was significantly increased with NTEM at 1.5 ml/L. Zn is a component of Zn metalloenzymes, which preserve the structural integrity of proteins, and may be responsible for these improvements in the serum biochemistry of rabbits [5].

NTEM supplementation in water selectively affected hematological traits. The low NTEM level increased RBC and hemoglobin levels, while all levels increased monocyte percentages, suggesting improved oxygen transport and potential immunomodulatory effects. Basophils were reduced at the low NTEM level, indicating subtle changes in leukocyte composition. The obtained data showed that serum, Hb, and RBC values were in optimal status at the end of the study with NTEM treatment, except for (0.5 ml/L), which might be due to the iron being available in the NTEM combination administered to the rabbits. This research study’s findings are consistent with [37], who reported that when a combination of elements was added to the feed, all animals had the best possible iron status, with the optimum RBC, Hb, and MCV levels for their age and BW.

NTEM supplementation in drinking water influenced serum protein fractions in growing rabbits. The high NTEM level increased albumin concentration, while the low and medium levels reduced it compared to the control, indicating a dose-dependent effect on hepatic protein synthesis. No significant changes were observed in α-, β-, or γ-globulins, suggesting that NTEM selectively affected albumin without altering major globulin fractions. The increase in albumin with a concurrent decrease in globulin fractions in the 1.5 ml/L NTEM group may be linked to the influence of trace elements on hepatic protein synthesis and immune modulation. Trace elements support albumin production as a marker of improved nutritional and antioxidant status, while the reduction in globulins likely reflects a lower antigenic or inflammatory stimulus, indicating efficient immune regulation rather than suppression. Serum triglyceride concentration decreased significantly with all NTEM treatments compared to the control group; this may be related to manganese. Similar to our findings, Chen et al. [4] observed that as manganese was added to the diet, the serum triglyceride levels of rex rabbits gradually decreased. This explanation may also account for the lower concentration of harmful cholesterol (LDL) and the higher concentration of good cholesterol (HDL) observed with NTEM treatments compared to the control group. In addition, zinc supplementation may be associated with decreases in serum triglycerides and HDL levels because it inhibits lipolysis in adipose tissue, leading to the breakdown of triglycerides stored in fat cells and the release of fatty acids and glycerol [9]. NTEM supplementation in drinking water had selective effects on lipid metabolism in growing rabbits. While total lipids and cholesterol remained unchanged, medium NTEM reduced triglycerides, high NTEM increased HDL, and low to medium NTEM decreased LDL, indicating a modulatory effect on lipoprotein fractions without altering overall lipid content. These study findings confirmed those observed by [1], who reported a significantly reduced serum cholesterol concentration in rabbits supplemented with ZnO nanoparticles.

In the current study, no significant changes were observed in internal organs, except for NTEM treatment (0.5 ml/L), which increased liver weight compared with the control group. Our findings are consistent with [16], who observed that the tested supplementation treatments had no appreciable impact on the carcass, liver, kidney, and head and hind portion weights. Additionally, carcass characteristics did not change in rabbits fed diets supplemented with 50, 100, 200, or 400 mg of zinc oxide per kg of diet [22].

## Conclusion

In conclusion, supplementation of drinking water with varying levels of NTEM improved growth performance, feed efficiency, and certain hematological and biochemical parameters in growing rabbits, without adverse effects on carcass traits. These findings suggest that NTEM could be a promising, environmentally friendly mineral supplement for rabbit production, although further studies under different management and environmental conditions are recommended to confirm its broader applicability.

## Data Availability

The data used to support our study’s findings are included in the article, and data coding is available from the corresponding author upon reasonable request.
